# Identification of a Guanine Nucleotide Exchange Factor for Arf3, the Yeast Orthologue of Mammalian Arf6

**DOI:** 10.1371/journal.pone.0000842

**Published:** 2007-09-05

**Authors:** Alison K. Gillingham, Sean Munro

**Affiliations:** Medical Research Council Laboratory of Molecular Biology, Cambridge, United Kingdom; University of Geneva, Switzerland

## Abstract

Small G proteins of the Arf and Rab families are fundamental to the organisation and activity of intracellular membranes. One of the most well characterised of these G proteins is mammalian Arf6, a protein that participates in many cellular processes including endocytosis, actin remodelling and cell adhesion. Exchange of GDP for GTP on Arf6 is performed by a variety of guanine nucleotide exchange factors (GEFs), principally of the cytohesin (PSCD) and EFA6 (PSD) families. In this paper we describe the characterisation of a GEF for the yeast orthologue of Arf6, Arf3, which we have named Yel1 (yeast EFA6-like-1) using yeast genetics, fluorescence microscopy and *in vitro* nucleotide exchange assays. Yel1 appears structurally related to the EFA6 family of GEFs, having an N-terminal Sec7 domain and C-terminal PH and coiled-coil domains. We find that Yel1 is constitutively targeted to regions of polarised growth in yeast, where it co-localises with Arf3. Moreover the Sec7 domain of Yel1 is required for its membrane targeting and for that of Arf3. Finally we show that the isolated Yel1 Sec7 domain strongly stimulates nucleotide exchange activity specifically on Arf3 *in vitro*.

## Introduction

Small G proteins of the Arf family control many aspects of intracellular membrane trafficking, interacting spatially and dynamically with a variety of proteins [1]. This adaptability is achieved because like most G proteins of the Ras superfamily, Arfs can cycle between GDP-bound and GTP-bound forms. This switching of nucleotide results in a large conformational change primarily to two regions of the protein, known as switch I and switch II. These regions are the main sites of interaction for downstream effectors as well as regulators of the G protein. The main regulators of the GDP-GTP cycle are the guanine nucleotide exchange factors (GEFs) that mediate the rate-limiting exchange of GDP for GTP, and the GTPase activating proteins (GAPs) that stimulate the hydrolysis of bound GTP.

Arf G proteins associate weakly with membranes in the GDP-bound form. Following GTP exchange the N-terminal amphipathic helix, which in most Arfs is also myristoylated, is exposed and can form a tight, stable interaction with the membrane [1]. This allows the G protein to recruit effector proteins to distinct organelles or sub-compartments within the cell. All Arf-GEFs described so far are peripheral membrane proteins that contain a region of approximately 200 amino acids that is responsible for their GEF activity and is known as the Sec7 domain [2]. Database analysis has revealed that there are 15 Sec7 domain containing proteins in humans, 8 in plants and 5 in flies, worms and yeast [3]. In mammals these proteins can be sub-divided into six families (BIG, GBF1, EFA6 (PSD), BRAGs (IQSEC), cytohesins (PSCD) and FBXO8) of which only the BIG and GBF1 families appear present in all eukaryotes [1], [4]. Regions outside the Sec7 domain are largely unrelated, however both EFA6 and cytohesin family members contain small regions of amphipathic helix and well conserved PH domains.

Two of the most well studied members of the Arf family are Arf1 and Arf6 [5]. Arf1 (and its close relatives Arfs 2-5) are chiefly responsible for regulating vesicle budding by recruiting protein complexes such as COPI, adaptor protein complex-1 and the GGAs to their sites of action [6]–[10]. Arf1 is activated *in vivo* by exchange factors of the GBF1 and BIG subfamilies [11]–[14]. In contrast Arf6 plays a role in actin cytoskeleton dynamics and endocytic processes, and localises in its GTP-bound form to the plasma membrane [5], [15], [16]. GTP-loading of Arf6 is stimulated by several families of GEFs and is presumably regulated by cellular context and via activation of signalling pathways in response to differing stimuli. Arf6 GEFs include BRAG (IQSEC) [17], [18], cytohesin (PSCD) [19], [20] and EFA6 (PSD) family members [21], [22].

Arf3 is the yeast orthologue of Arf6 [23]. Unlike its mammalian counterpart it has not been extensively studied, although recent data suggests that it too plays a role in regulating the actin cytoskeleton [24], [25]. In addition Arf3 may function in the development of cell polarity in yeast [26]. In this study we report the identification of a guanine nucleotide exchange factor for Arf3, which we have termed Yel1 (yeast EFA6-like-1) due to its overall domain architecture and sequence similarity to mammalian EFA6. We examine the intracellular localisation of Yel1 and analyse the specificity of its nucleotide exchange activity *in vitro*.

## Results and Discussion

### The gene YBL060w encodes a protein with an N-terminal Sec7 domain

**Figure 1:**
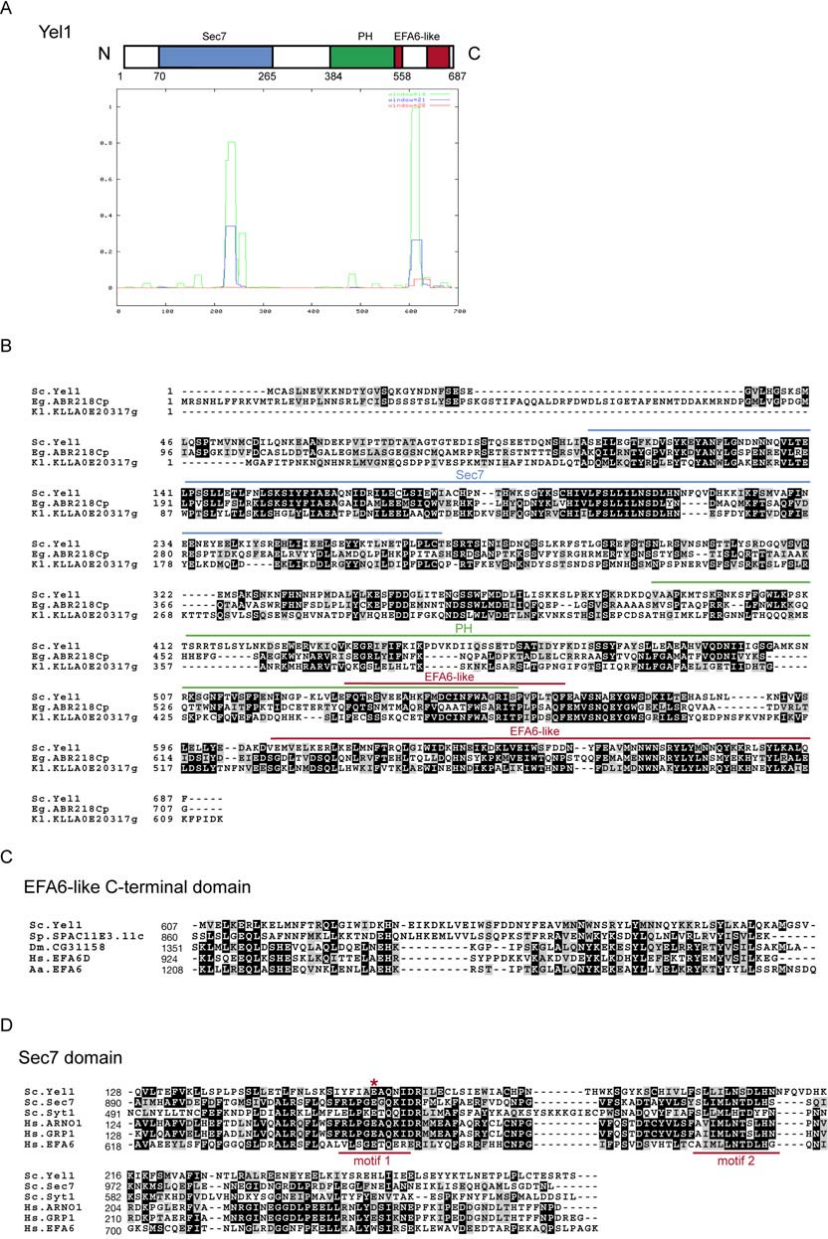
Yel1 is a member of a conserved family of Guanine Nucleotide Exchange Factors. (A) Schematic diagram of the domain structure of Yel1, showing the relative positions of the Sec7 domain, the PH domain and the EFA6-like regions. Also shown is a coiled-coil prediction for Yel1 [40]. (B) Alignment of Yel1 with its two closest relatives from Fungi. The sequences were aligned using CLUSTALW and shaded grey when more then half the residues are related and black when they are identical. The positions of the Sec7, the PH domain and regions related to the EFA6 family of GEFs are indicated. (C) Alignment of the second EFA6-like domain found in Yel1 (highlighted in (B)) with those from other species. The C-terminal 157 residues of Yel1 were used for a PSI-BLAST search (cut off E<0.005, and after one iteration found Drosophila EFA6 (CG31158, E = 4×10^−5^), and after two iterations found several mammalian EFA6s (E = 6×10^−10^). No members of the other mammalian Sec7 domain families were obtained, even after six iterations after which no further sequences were found above the threshold. (D) Alignment of the Sec7 domain from Yel1 with those from yeast Sec7 and Syt1 and human EFA6B (PSD4), ARNO (PSCD2) and GRP1 (PSCD3). The critical catalytic glutamate (E) is highlighted (red asterix). *Aa, A. aegypti; Eg, E. gossippi; Dm, D. melanogaster; Hs, H. sapiens; Kl, K. lactis; Sc, S. cerevisiae; Sp, S. pombe.*

The GEFs for a number of Arf and the related Arf-like (Arl) G proteins have yet to be determined. Previous studies have shown that the most recognisable characteristic of Arf-GEFs is the catalytic Sec7 domain, first identified in the yeast protein Sec7, a protein with a key role in secretion [19], [27]. Using this domain and the iterative search programme PSI-BLAST to mine the GenBank database revealed the presence of an uncharacterised protein in yeast encoded by the gene YBL060w. This protein has 687 amino acids, with a Sec7 domain located near to the N-terminus (residues 70–265). A small region of the YBL060w gene product, like the EFA6 and cytohesin GEFs, is predicted to form an amphipathic helix and there is a PH domain (residues 384–558) in the C-terminal half of the protein (Figure 1A and 1B). Indeed a recent study analysing the specificity of all the PH domains in yeast reported that the YBL060w gene product bound phosphoinositides, although this binding was relatively weak and rather promiscuous [28].

The sequence of the YBL060w gene product is not especially well conserved in evolution with its closest relatives, uncharacterised proteins from the budding yeast *E. gosspyii* (ABR218Cp) and *K. lactis* (KLLA0E20317g), sharing about 30% sequence identity (Figure 1B). However there are clear orthologues in other yeasts and filamentous fungi. Moreover, iterative PSI-BLAST with the C-terminal portion of Yel1 reveals two regions that are related to the EFA6 family of mammalian Arf6 GEFs, one being the PH domain and in particular a region at its C-terminal end and the other being at the extreme C-terminus of the protein (Figure 1A–C). Furthermore, the overall size and domain architecture of the YBL060w gene product resembles the EFA6 exchange factors. Thus we have named the protein Yel1 for yeast EFA6-like-1.

Alignment of the Yel1 Sec7 domain with those from other GEFs in yeast (Sec7, Syt1) and humans (ARNO, GRP1 and EFA6B) demonstrates that two highly conserved regions, known from crystallography studies of GEF:G protein complexes to combine to form the active site, are also present in Yel1 (motifs 1 and 2, Figure 1D). Indeed the crystal structure of yeast Gea2-Sec7 bound to human Arf1 reveals that these 2 regions border a hydrophobic groove into which residues from switch I and switch II of the G protein insert [29]. Moreover, all Arf-GEFS share a conserved glutamate residue known to be critical for catalysis. This invariant residue, which acts to promote nucleotide dissociation by inserting into the nucleotide-binding pocket of the G protein [29], is also conserved in Yel1 (Figure 1D).

### Yel1 is required for the localisation of Arf3 to the plasma membrane

**Figure 2:**
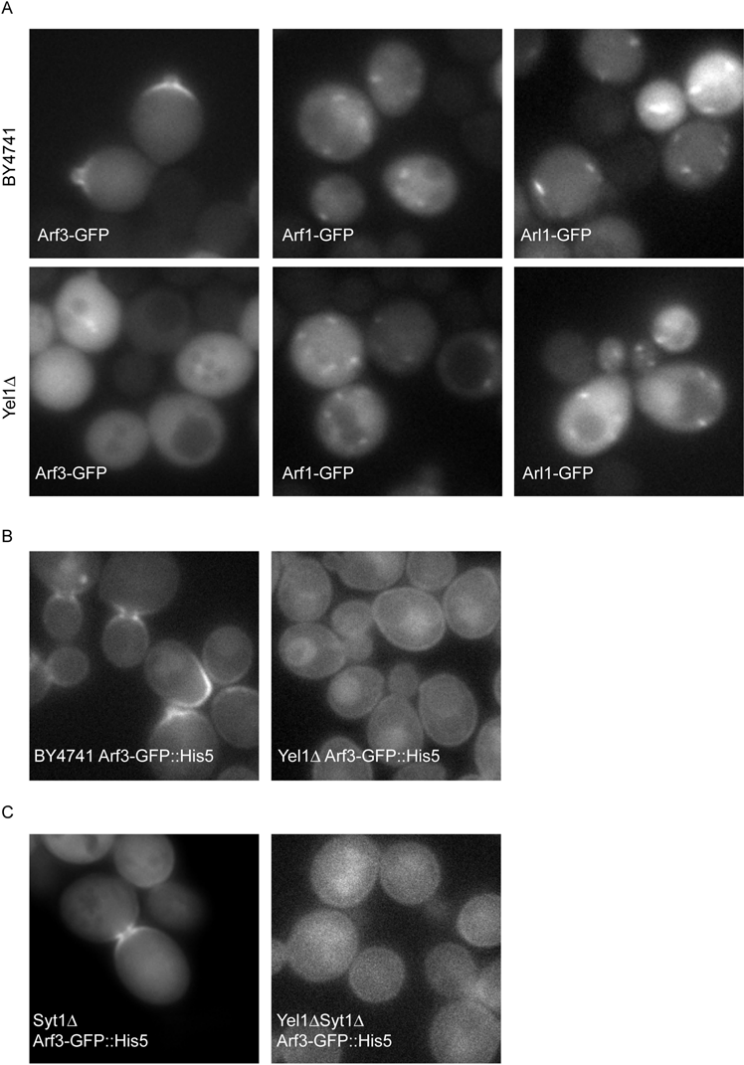
Yel1 is required for the localisation of Arf3. (A) Fluorescent micrographs of live yeast expressing the indicated fusion proteins. Arfs and Arls were tagged at the C-terminus and expressed from a *PHO5* promotor in both a wild-type (BY4741, top panel) and a *yel1*Δ strain (lower panel). Only the distribution of Arf3 was affected by loss of Yel1. (B) Arf3 tagged in the genome of wild-type (BY4741), and *yel1*Δ mutant strains under its own promotor. Under these conditions loss of Yel1 results in an increase in cytoplasmic Arf3 as in (A). In addition some weak plasma membrane fluorescence is apparent, although this is no longer restricted to sites of polarisied growth, and is not affected by additional loss of Syt1 (C).

Arfs and Arls are recruited to membranes in their active, GTP-bound form. To determine whether Yel1 is a GEF for a known G protein we exploited the fact that *YEL1* is a non-essential gene in yeast. We reasoned that, unless there is redundancy between Yel1 and another GEF, loss of the protein will result in the mislocalisation of its target G protein. Thus we expressed plasmid-borne copies of GFP tagged Arf1, Arf3, Arl1 or Arl3 in both wild-type and *yel1Δ* mutant strains. As expected in wild-type cells Arf1, Arl1 (Figure 2A) and Arl3 (data not shown) all localised to punctate structures scattered throughout the cell, which are typical of the Golgi. This distribution was unaffected by loss of Yel1. In contrast Arf3, which in the wild-type strain targeted to the bud neck and bud tip, was found localised throughout the cytoplasm in a *yel1Δ* mutant strain (Figure 2A).

To confirm these observations in the absence of over-expression, strains were created in which Arf3 was tagged at the C-terminus in the genome of wild-type or *yel1Δ* mutant yeast. Under these conditions the G protein is expressed from its own promotor. As before Arf3 localised to regions of polarised growth in the wild-type strain. When Yel1 was absent the majority of Arf3 was displaced to the cytoplasm however a weak plasma membrane localisation was also observed in the majority of cells. Strikingly, residual targeting of Arf3 to the membrane under these conditions was no longer restricted to the emerging bud but instead appeared uniformly distributed around the plasma membrane (Figure 2B). Finally we tested whether the fungal specific Sec7-domain protein Syt1, (which is also encoded by a non-essential gene in yeast), could function in the Arf3 pathway. This GEF has been reported to exhibit some weak nucleotide exchange activity towards Arf2, stimulating the release of [^3^H]GDP approximately 2-fold, however its activity on Arf3 has not been reported [30]. Figure 2C shows that loss of Syt1 had no effect on the localisation of Arf3 in the presence or absence of Yel1.

### Yel1 and Arf3 co-localise at sites of polarised growth on the plasma membrane

**Figure 3:**
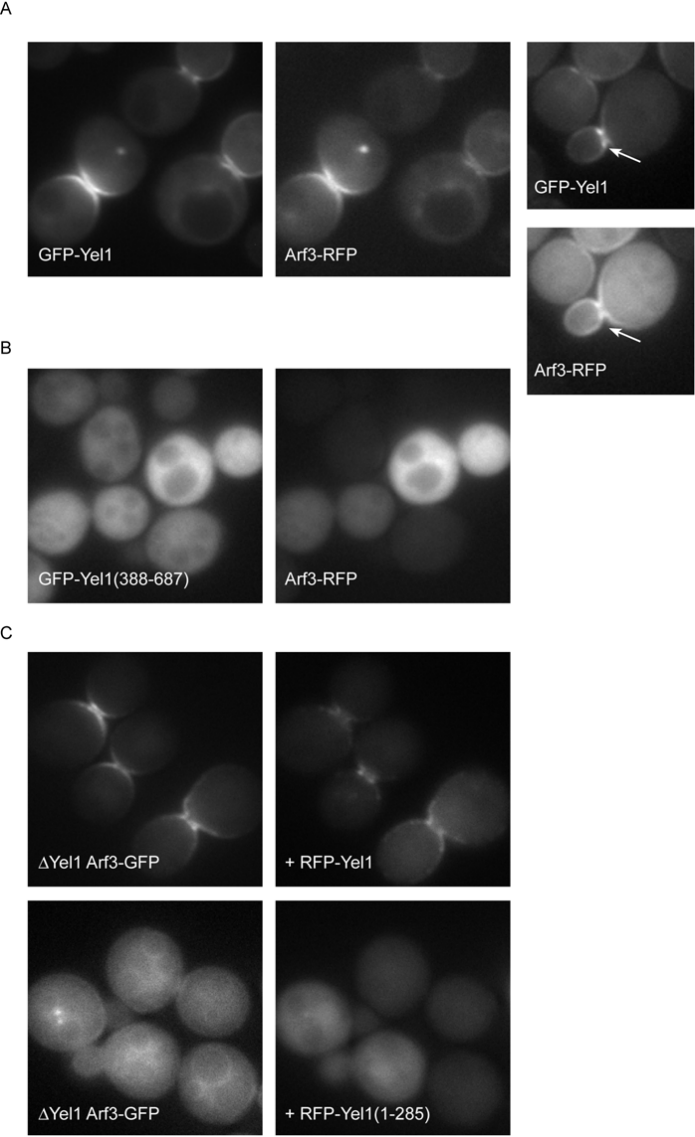
The Sec7 domain of Yel1 is required for its localisation and for the localisation of Arf3. Fluorescent micrographs of live yeast expressing the indicated fusion proteins. (A) Yel1 was tagged with GFP in the genome of an *arf3*Δ strain, whereas Arf3-RFP was expressed in the same cells from a *CEN* plasmid under the control of a *PHO5* promotor. The two proteins were found to co-localise both to regions of polarised growth and to occasional intracellular structures. At the bud neck and bud tip the distribution of Yel1 was more restricted than that of Arf3 (arrows, right hand panels). (B) Loss of the N-terminal portion of the Yel1 protein (GFP-Yel1(388-687) resulted in its redistribution to the cytoplasm, along with Arf3-RFP. (C) Expression of the Sec7 domain of Yel1 (RFP-Yel1(1-285)) failed to rescue the mislocalisation of Arf3-GFP in a *yel1*Δ strain.

To characterise Yel1 in more detail we next asked whether its localisation was consistent with the targeting of Arf3 to the bud neck. Yel1 was initially C-terminally tagged in the genome of an *arf3Δ* mutant strain with GFP and expressed under its own promotor. The resulting Yel1-GFP fusion localised to the bud, however the fluorescence signal was weak negating the possibility of two-colour imaging with Arf3-RFP. To circumvent this problem the fusion protein was re-engineered so that it could be overexpressed from a *PHO5* promotor with an N-terminal GFP tag. This construct was clearly visible at the plasma membrane where, like the C-terminal fusion, the majority was restricted to the bud neck and the emerging bud tip. As expected, co-expressed Arf3-RFP co-localised extensively with Yel1 at the plasma membrane (Figure 3A), implying that the tagged Yel1 protein is functional. Moreover we occasionally observed that the localisation of Yel1 was more tightly restricted to the bud neck and bud tip than that of Arf3, particularly when Arf3 was expressed at high levels (right hand panels). One possible explanation for this is that following nucleotide exchange Arf3 can diffuse away from its site of activation before being turned over and released from the membrane. In addition we also observed 1–2 intracellular punctate structures that contained both Arf3 and Yel1 in approximately 10% of budding cells. The exact nature of these organelles is unclear, but they may represent endocytic compartments.

### The N-terminal domain of Yel1 is required for its own localisation and for that of Arf3

Some members of the cytohesin and EFA6 families of Arf-GEFs are stabilised on their target membranes by the contribution of their PH domains, which interact with specific phosphoinositides [31], [32]. To determine whether Yel1 can also localise to the membrane in this manner, a strain was constructed in which residues 1–387 were replaced with GFP in the genome of an *arf3Δ* mutant. This truncation removes the Sec7 domain leaving only the C-terminal portion of the protein that contains the PH domain. At steady state GFP-Yel1(388-687) was undetectable at the plasma membrane and was found instead throughout the cytoplasm (Figure 3B). These data suggest that, although Yel1 contains a recognisable PH domain, its affinity for phosphoinositides is insufficient to allow the protein to stably associate with the plasma membrane. Interestingly a study by Mark Lemmons' group in which the activities of all PH domains from yeast were assessed using a Ras activation assay demonstrated that, under certain conditions, a Ras-Yel1-PH domain fusion was capable of targeting Ras to the membrane, where it could rescue the defect of a *cdc25^ts^* mutant strain [28]. However when this construct was tagged with GFP and localised in the cell it was not found at the membrane but instead targeted to the nucleus. These observations suggest that the PH domain of Yel1 is able to associate weakly with membranes but that the degree of membrane association is undetectable at the level of the light microscope. The results also imply that in the full-length protein, the N-terminus, perhaps through the activity of the Sec7 domain, is additionally required to ensure that a stable interaction with the membrane is sustained. We next examined the localisation of Arf3 in the GFP-Yel1(388-687) mutant yeast. As expected, Arf3-RFP was displaced from the plasma membrane in this strain (Figure 3B).

Since the isolated C-terminal portion of Yel1 is not capable of stably localising the protein to the membrane, we next asked whether the Sec7 domain can perform this function. To address this, a *yel1Δ* mutant strain in which endogenous Arf3 is tagged with GFP was transformed with plasmid-borne copies of RFP-Yel1 or RFP-Yel1(1-285), the latter plasmid encoding only the Sec7 domain. As expected full-length Yel1 targeted to the plasma membrane and could rescue the mislocalisation of Arf3-GFP observed in the *yel1Δ* strain (Figures 3C and 2B). In contrast, although the Sec7 domain of Yel1 can promote nucleotide exchange on Arf3 (see later), expression of the Sec7 domain alone could not re-localise Arf3 to regions of polarised growth. Instead both RFP-Yel1(1-285) and Arf3-GFP were found distributed throughout the cytoplasm (Figure 3C), (although in the case of Arf3-GFP some weak non-polarised association with the plasma membrane was apparent as is also observed in the *yel1Δ* strain (Figure 2B)). Thus the Sec7 domain of Yel1 is neither sufficient for its own membrane recruitment nor for that of Arf3.

To analyse the targeting of Yel1 in more detail two additional truncation mutants were engineered, RFP-Yel1(1-371) and RFP-Yel1(1-556) and transformed into the above strain. Neither of these truncated proteins proved particularly stable in cells and both failed to rescue the mislocalisation of Arf3 (data not shown). Moreover we mutated several conserved residues in the PH domain and C-terminal EFA6-like region of RFP-Yel1 (single mutants F540A and W569A and triple mutant K506A, R507A, K508A) in order to isolate a Yel1 protein that could no longer target to the membrane. However in all cases the Yel1 mutant behaved as wild-type protein and could rescue the Arf3 targeting phenotype. Together our results suggest that targeting information for Yel1 is encoded throughout the length of the protein.

### The guanine nucleotide exchange activity of Yel1 is specific for Arf3

In all cases so far examined, the catalytic activity of Arf GEFs has been attributed to the Sec7 domains of these proteins [11], [14], [19]. Thus we sought to determine whether the isolated Sec7 domain from Yel1 could display nucleotide exchange activity towards Arf3. To do this we used an *in vitro* nucleotide exchange assay to compare the activity of the Yel1 Sec7 domain towards a number of small G proteins [33]. Recombinant yeast Arf1, Arf3 or Arl1 were all expressed as GST fusion proteins. In each case the first 14 amino acids, which form an amphipathic helix involved in membrane stabilisation, were removed, since this has been shown to allow nucleotide exchange activity to be monitored in the absence of liposomes [34]. Following isolation of the G proteins on glutathione Sepharose and removal of the GST tag, the purified proteins were loaded with GDP as described in Materials and Methods.

**Figure 4:**
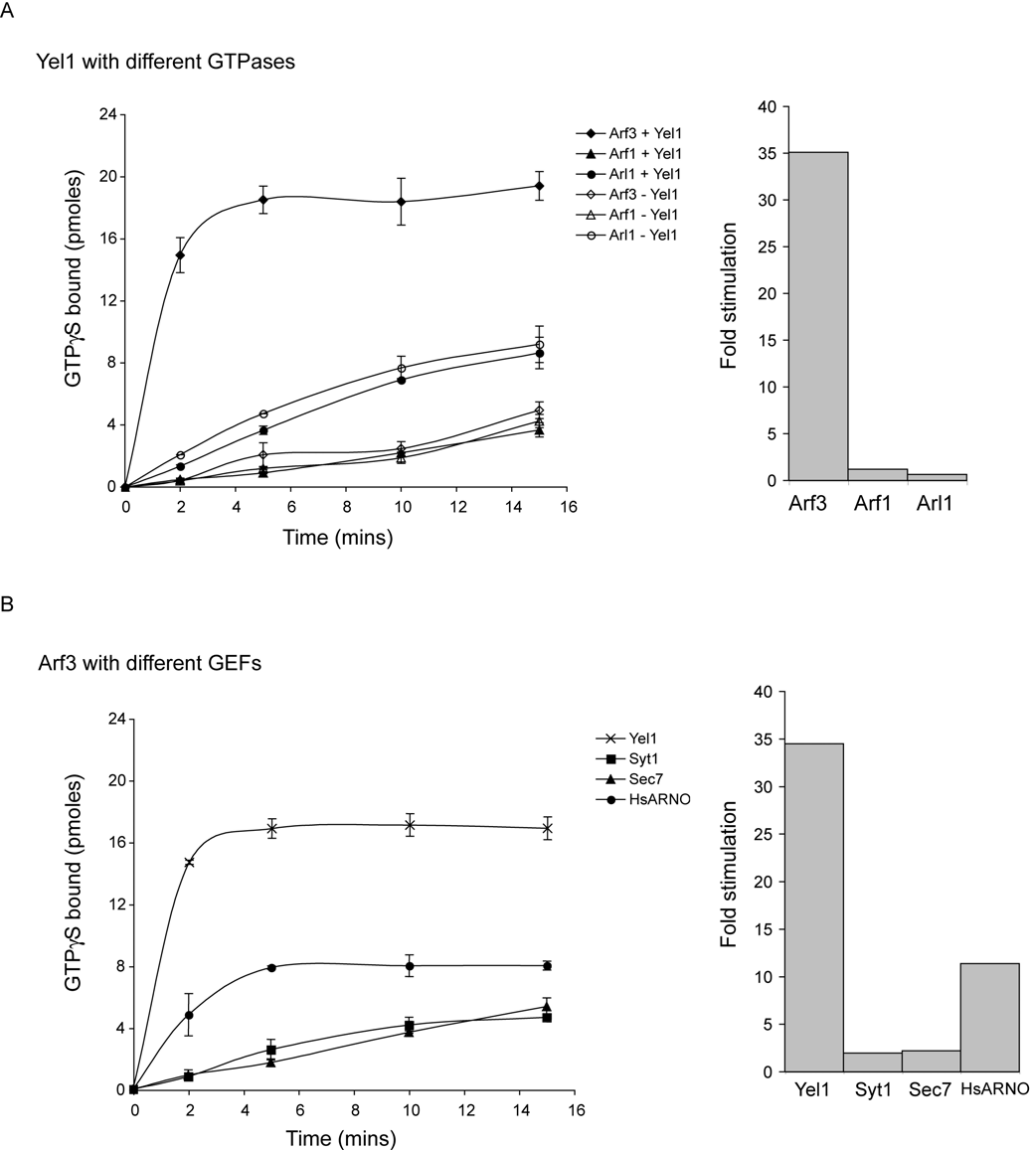
Yel1 stimulates guanine nucleotide exchange on Arf3. (A) Yel1 preferentially stimulates nucleotide exchange on Arf3. The kinetics of GDP to GTP exchange on Arfs were monitored by determining the binding of [^35^S]GTPγS to 2.5 µM rArf1, rArf3 or rArl1 in the absence or presence of 50 nM Yel1 Sec7 domain as described in Materials and Methods. Fold stimulation (right hand panel) is shown as the difference in GDP to GTP exchange at 2 mins+ and –GEF. It should be noted that the rate of spontaneous exchange on Arl1 is higher than that of Arf1 or Arf3, perhaps implying that, at least *in vitro,* Arl1 has a lower intrinsic affinity for nucleotides than either of the other G protein. Since the N-terminal amphipathic helix is known to play a role in “locking” G proteins in their GDP-bound conformation, it may be that removal of this domain from Arl1 has a more stimulatory effect on spontaneous nucleotide exchange than the same deletion from Arf1 or Arf3. (B) Comparison of the efficiency of Sec7 domains from different GEFs to stimulate GDP to GTP exchange on Arf3. Exchange on rArf3 was monitored as in (A) with Sec7 domains from Yel1 (cross symbols), Syt1 (squares), Sec7 (triangles) and human ARNO (circles) added at a final concentration of 50 nM. Fold stimulation (right hand panel) is shown as the difference in GDP to GTP exchange at 2 mins+ and –the different GEF proteins. Values are the means of two or three independent experiments performed in duplicate and were converted to pmoles by determining the CPM/pmole of GTPγS for each experiment. The maximum binding corresponded to ≈15–20% of the concentration of rArf3 used.

The catalytic activity of the Yel1 Sec7 domain can be expressed as the fold stimulation over the spontaneous exchange activity of the G protein. Thus we monitored nucleotide exchange on Arf3 in the presence of Yel1 Sec7 after 2 min incubation with the GEF. Recombinant Yel1 Sec7 domain at a final concentration of 50 nM stimulated nucleotide exchange on Arf3 nearly 35-fold. The same concentration of GEF had little effect on the rate of nucleotide exchange by Arf1 or Arl1 (Figure 4A).

We next tested whether other yeast GEFs were able to stimulate nucleotide exchange on Arf3 *in vitro*. To this end the Sec7 domains of Syt1 and Sec7 were expressed and purified from *E. coli*. As before we analysed the fold stimulation in exchange activity of the isolated Sec7 domains over spontaneous exchange on Arf3. Under the conditions used here neither yeast protein could significantly stimulate nucleotide exchange on Arf3 (Figure 4B). In contrast the Sec7 domain from yeast Sec7 promoted efficient nucleotide exchange on Arf1, confirming that it was properly folded (data not shown).

Finally we tested the activity of human ARNO, a GEF known to stimulate nucleotide exchange on Arf6 [35]. In contrast to Sec7 and Syt1, human ARNO stimulated nucleotide exchange on yeast Arf3 by approximately 11-fold (Figure 4B), indicating that although Yel1 and ARNO are only distantly related at the sequence level, they may share a conserved mode of action towards the Arf6 family of small G proteins that is not shared by other Arf GEFs.

In summary this paper describes the identification of Yel1 as a new guanine nucleotide exchange factor in yeast. This GEF is specific for yeast Arf3, a small plasma membrane localised G protein orthologous to the well-studied mammalian protein, Arf6. Over the past several years the number of exchange factors for Arf6 has been steadily growing and the list now includes GEFs from the BRAG (IQSEC), cytohesin (PSCD) and EFA6 (PSD) families. Why there are such a diverse set of exchange factors for Arf6 and how they regulate a single small G protein remain open questions. Moreover the relative contribution of each of these GEFs towards Arf6 function is complicated by the fact that most experiments are performed in the presence of other Arf6-specific GEFs. Regions of Yel1, particularly in the C-terminal half of the protein, share significant homology with EFA6. This suggests that the EFA6/Yel1 family of GEFs are more evolutionarily ancient Arf6 exchange factors than the other families found in mammals. Thus the identification of a single exchange factor for Arf3 may provide a useful tool for examining EFA6-like proteins in the absence of competing GEFs.

Arf3 and Yel1 co-localise in the emerging bud. However upon loss of Yel1, Arf3 is not only mis-localised to the cytosol but also loses its polarised distribution and is, instead, dissipated weakly around the plasma membrane. It is tempting to speculate that the low levels of residual membrane bound Arf3-GFP observed in the *yel1Δ* strain represent an inactive form of the protein which is only loosely associated with the membrane and that it is the presence of the GEF that recruits Arf3-GFP into the bud. Indeed a recent paper reported that a GDP-locked, inactive mutant of human Arf6 also targets to the cell surface, leading the authors to suggest that GDP/GTP exchange on Arf6 occurs after the protein has bound to the plasma membrane [22].

The precise role of Arf3 in yeast is, at present, unclear. A direct link between Arf3 and a component of the endocytic machinery, Lsb5, has recently been reported [36]. Moreover Arf3 has been linked to actin cable and cortical patch formation [25], [37]. Others have argued against a role in endocytosis and instead implicate Arf3 solely in the establishment of cell polarity [26]. Neither role need be mutually exclusive but since the function of Arf3 remains controversial we can only speculate as to the role of Yel1. As mentioned above since the distribution of Arf3 reflects the distribution of Yel1, the simplest function for Yel1 would be to localise and stimulate GTP-binding on Arf3 in the correct place for it to perform its tasks. This implies that there must be mechanisms in place to ensure a polarised distribution of Yel1. These interactions may well be stabilised by the contributions of the PH domain and C-terminal coiled-coil region but clearly our data shows that additional targeting information is required. It will be important to determine precisely how Yel1 is targeted to the emerging bud, and as such how this regulates the polarisation of Arf3 at this site.

## Materials and Methods

### Yeast strains and *media*

All yeast strains were based on BY4741 (*MATa his3*Δ*1 leu2*Δ*0 met15*Δ*0 ura3*Δ*0*) or deletion strains within this background from the EUROSCARF consortium (Table 1). Tagging and truncations of open reading frames (ORFs) at the N-terminus and C-terminus were performed using PCR-based homologous recombination [38]. Other tagged proteins were expressed from pRS series CEN plasmids under the control of a constitutive PHO5 promoter unless otherwise stated. Arf3, Arf1 and Arl1 were tagged at the C-terminus with GFP and RFP (DsRed (tdimer2)[39]) with a GAGAGA linker between the GFP or RFP cassette and the end of the ORF.

### Preparation of recombinant G proteins

Arf1, Arf3 or Arl1 lacking the N-terminal 14 residues were expressed with GST fused to their N-termini by using the plasmid pGEX-6p-2 (Amersham Biosciences). *E. coli* BL21-GOLD (DE3) cells (Stratagene) were induced at OD_600_≈0.7 with 0.2 mM isopropyl-β-thiogalactopyranoside (IPTG) and incubated overnight at 17°C. Lysates were prepared by sonication in 20 ml of lysis buffer (50 mM HEPES-KOH, pH 7.5, 100 mM KCl, 100 µM GDP, 5 mM EDTA, 1 mM MgCl_2_, 1 mM DTT) containing protease inhibitors. The lysates were clarified by centrifugation at 12,000×*g* for 10 min. GST-Arf1, GST-Arf3 or GST-Arl1 were isolated on glutathione Sepharose beads (Amersham Biosciences) at 4°C for 30 min, then released from the beads and the GST tag using Prescission protease (Amersham Biosciences). Untagged G proteins were incubated with 100 µM GDP for 45 min at 37°C to ensure that all the G protein was in the GDP bound state and this was stabilised by the addition of 10 mM MgCl_2_. G proteins were then loaded onto PD-10 columns pre-equilibrated in stabilisation buffer (50 mM HEPES-KOH, pH 7.5, 100 mM KCl, 5 mM EDTA, 10 mM MgCl_2_, 1 mM DTT) to remove unbound GDP. Fractions were collected, analysed for the presence of the G protein by Coomassie staining of nitrocellulose dot blots and pooled. Purified G proteins were stored in 50% glycerol at −20°C.

### Preparation of recombinant GEFs

Sec7 domains from yeast Yel1 (S91-S275), Sec7 (S807-G1021), and human ARNO (M1-D261) were tagged N-terminally with a hexa-histidine tag in the vector pOPTH (Met-Ala-His_6_) generously provided by O. Perisic (MRC-LMB, Cambridge, U.K.). Syt1 (S447-L635) was tagged C-terminally with hexa-histidine using the pET24b vector (Novagen). Sec7 domains were prepared from bacteria induced, lysed and clarified by centrifugation as described for G proteins (see above). His_6_-tagged proteins were then purified using the His-Bind® purification kit (Calbiochem) according to the manufacturers instructions. Purified proteins were dialysed overnight against GEF assay buffer (50 mM HEPES-KOH, pH 7.5, 100 mM KCl, 1 mM MgCl_2_, but without DTT), then stored in 50% glycerol at −20°C.

### Nucleotide binding assays

G proteins (2.5 µM) were incubated at 28°C with [^35^S]GTPγS (50 µM, ≈800 CPM/pmol) in GEF assay buffer (50 mM HEPES-KOH, pH 7.5, 100 mM KCl, 1 mM MgCl_2_, 1 mM DTT). Unless otherwise stated GEFs were added at a final concentration of 50 nM. Samples of 5 µl were removed at various time points, diluted with 2 ml ice-cold stop buffer (50 mM HEPES-KOH, pH 7.5, 100 mM KCl, 10 mM MgCl_2_, 1 mM DTT), and filtered on 25 mm BA-85 nitrocellulose filters. Filters were washed three times in the same buffer, dried and counted in a liquid scintillation counter (Beckman, LS6000SC).

### Yeast microscopy

Live yeast expressing GFP and RFP fusions were photographed under coverslips with a Zeiss Axioscop and a CCD camera (Princeton Instruments, Trenton NJ) using 0.5–1 second exposures.

**Table 1:** Yeast strains used in this study:

Name	Genotype
AGY72	BY4741 GFP-*Arf3::Sp.his5*
AGY73	BY4741 *yel1*Δ*::kanMX4, Arf3*-GFP*::Sp.his5*
AGY74	BY4741 *yel1*Δ*::kanMX4, syt1*Δ*:: NatMX4, Arf3*-GFP*::Sp.his5*
AGY75	BY4741 *yel1*Δ*::kanMX4, gea2*Δ*:: NatMX4, Arf3*-GFP*::Sp.his5*
AGY76	BY4741 *arf3*Δ*::kanMX4*, GFP-*Yel1::Sp.his5*
AGY77	BY4741 *arf3*Δ*::kanMX4*, GFP-*Yel1ΔN*388*::Sp.his5*

## References

[1] Gillingham AK, Munro S (2007). The Small G Proteins of the Arf Family and Their Regulators.. Annu Rev Cell Dev Biol (in press).

[2] Shin HW, Nakayama K (2004). Guanine nucleotide-exchange factors for arf GTPases: their diverse functions in membrane traffic.. J Biochem (Tokyo).

[3] Cox R, Mason-Gamer RJ, Jackson CL, Segev N (2004). Phylogenetic analysis of Sec7-domain-containing Arf nucleotide exchangers.. Mol Biol Cell.

[4] Kahn RA (2004). ARF Family GTPases, Vol. 1: Kluwer Academic Publishers, Dordrecht..

[5] D'Souza-Schorey C, Chavrier P (2006). ARF proteins: roles in membrane traffic and beyond.. Nat Rev Mol Cell Biol.

[6] Williger BT, Ostermann J, Exton JH (1999). Arfaptin 1, an ARF-binding protein, inhibits phospholipase D and endoplasmic reticulum/Golgi protein transport.. FEBS Lett.

[7] Nickel W, Malsam J, Gorgas K, Ravazzola M, Jenne N (1998). Uptake by COPI-coated vesicles of both anterograde and retrograde cargo is inhibited by GTPgS in vitro.. J Cell Sci.

[8] Donaldson JG, Cassel D, Kahn RA, Klausner RD (1992). ADP-ribosylation factor, a small GTP-binding protein, is required for binding of the coatomer protein beta-COP to Golgi membranes.. Proc Natl Acad Sci U S A.

[9] Shinotsuka C, Waguri S, Wakasugi M, Uchiyama Y, Nakayama K (2002). Dominant-negative mutant of BIG2, an ARF-guanine nucleotide exchange factor, specifically affects membrane trafficking from the trans-Golgi network through inhibiting membrane association of AP-1 and GGA coat proteins.. Biochem Biophys Res Commun.

[10] Volpicelli-Daley LA, Li Y, Zhang CJ, Kahn RA (2005). Isoform-selective effects of the depletion of ADP-ribosylation factors 1-5 on membrane traffic.. Mol Biol Cell.

[11] Peyroche A, Paris S, Jackson CL (1996). Nucleotide exchange on ARF mediated by yeast Gea1 protein.. Nature.

[12] Kawamoto K, Yoshida Y, Tamaki H, Torii S, Shinotsuka C (2002). GBF1, a guanine nucleotide exchange factor for ADP-ribosylation factors, is localized to the cis-Golgi and involved in membrane association of the COPI coat.. Traffic.

[13] Togawa A, Morinaga N, Ogasawara M, Moss J, Vaughan M (1999). Purification and cloning of a brefeldin A-inhibited guanine nucleotide-exchange protein for ADP-ribosylation factors.. J Biol Chem.

[14] Morinaga N, Tsai SC, Moss J, Vaughan M (1996). Isolation of a brefeldin A-inhibited guanine nucleotide-exchange protein for ADP ribosylation factor (ARF) 1 and ARF3 that contains a Sec7-like domain.. Proc Natl Acad Sci U S A.

[15] Cavenagh MM, Whitney JA, Carroll K, Zhang C, Boman AL (1996). Intracellular distribution of Arf proteins in mammalian cells. Arf6 is uniquely localized to the plasma membrane.. J Biol Chem.

[16] Donaldson JG (2003). Multiple roles for Arf6: sorting, structuring, and signaling at the plasma membrane.. J Biol Chem.

[17] Someya A, Sata M, Takeda K, Pacheco-Rodriguez G, Ferrans VJ (2001). ARF-GEP(100), a guanine nucleotide-exchange protein for ADP-ribosylation factor 6.. Proc Natl Acad Sci U S A.

[18] Dunphy JL, Moravec R, Ly K, Lasell TK, Melancon P (2006). The Arf6 GEF GEP100/BRAG2 regulates cell adhesion by controlling endocytosis of b1 integrins.. Curr Biol.

[19] Chardin P, Paris S, Antonny B, Robineau S, Beraud-Dufour S (1996). A human exchange factor for ARF contains Sec7- and pleckstrin-homology domains.. Nature.

[20] Langille SE, Patki V, Klarlund JK, Buxton JM, Holik JJ (1999). ADP-ribosylation factor 6 as a target of guanine nucleotide exchange factor GRP1.. J Biol Chem.

[21] Franco M, Peters PJ, Boretto J, van Donselaar E, Neri A (1999). EFA6, a sec7 domain-containing exchange factor for ARF6, coordinates membrane recycling and actin cytoskeleton organization.. Embo J.

[22] Macia E, Luton F, Partisani M, Cherfils J, Chardin P (2004). The GDP-bound form of Arf6 is located at the plasma membrane.. J Cell Sci.

[23] Huang CF, Buu LM, Yu WL, Lee FJ (1999). Characterization of a novel ADP-ribosylation factor-like protein (yARL3) in *Saccharomyces cerevisiae*.. J Biol Chem.

[24] Costa R, Ayscough KR (2005). Interactions between Sla1p, Lsb5p and Arf3p in yeast endocytosis.. Biochem Soc Trans.

[25] Lambert AA, Perron MP, Lavoie E, Pallotta D (2007). The Saccharomyces cerevisiae Arf3 protein is involved in actin cable and cortical patch formation.. FEMS Yeast Res.

[26] Huang CF, Liu YW, Tung L, Lin CH, Lee FJ (2003). Role for Arf3p in development of polarity, but not endocytosis, in *Saccharomyces cerevisiae*.. Mol Biol Cell.

[27] Achstetter T, Franzusoff A, Field C, Schekman R (1988). *SEC7* encodes an unusual, high molecular weight protein required for membrane traffic from the yeast Golgi apparatus.. J Biol Chem.

[28] Yu JW, Mendrola JM, Audhya A, Singh S, Keleti D (2004). Genome-wide analysis of membrane targeting by S. cerevisiae pleckstrin homology domains.. Mol Cell.

[29] Goldberg J (1998). Structural basis for activation of ARF GTPase: mechanisms of guanine nucleotide exchange and GTP-myristoyl switching.. Cell.

[30] Jones S, Jedd G, Kahn RA, Franzusoff A, Bartolini F (1999). Genetic interactions in yeast between Ypt GTPases and Arf guanine nucleotide exchangers.. Genetics.

[31] Klarlund JK, Guilherme A, Holik JJ, Virbasius JV, Chawla A (1997). Signaling by phosphoinositide-3,4,5-trisphosphate through proteins containing pleckstrin and Sec7 homology domains.. Science.

[32] Jackson TR, Kearns BG, Theibert AB (2000). Cytohesins and centaurins: mediators of PI 3-kinase-regulated Arf signaling.. Trends Biochem Sci.

[33] Franco M, Chardin P, Chabre M, Paris S (1995). Myristoylation of ADP-ribosylation factor 1 facilitates nucleotide exchange at physiological Mg2+ levels.. J Biol Chem.

[34] Beraud-Dufour S, Robineau S (2001). Expression, purification, and measurements of activity of ARNO1, a guanine nucleotide exchange factor for ADP-ribosylation factor 1 (ARF1).. Methods Enzymol.

[35] Frank S, Upender S, Hansen SH, Casanova JE (1998). ARNO is a guanine nucleotide exchange factor for ADP-ribosylation factor 6.. J Biol Chem.

[36] Costa R, Warren DT, Ayscough KR (2005). Lsb5p interacts with actin regulators Sla1p and Las17p, ubiquitin and Arf3p to couple actin dynamics to membrane trafficking processes.. Biochem J.

[37] Zakrzewska E, Perron M, Laroche A, Pallotta D (2003). A role for GEA1 and GEA2 in the organization of the actin cytoskeleton in Saccharomyces cerevisiae.. Genetics.

[38] Wach A, Brachat A, Alberti-Segui C, Rebischung C, Philippsen P (1997). Heterologous HIS3 marker and GFP reporter modules for PCR-targeting in Saccharomyces cerevisiae.. Yeast.

[39] Campbell RE, Tour O, Palmer AE, Steinbach PA, Baird GS (2002). A monomeric red fluorescent protein.. Proc Natl Acad Sci U S A.

[40] Lupas A (1996). Prediction and analysis of coiled-coil structures.. Methods Enzymol.

